# Deep Eutectic Solvents Based on Natural Ascorbic Acid Analogues and Choline Chloride

**DOI:** 10.1002/open.202000020

**Published:** 2020-05-04

**Authors:** Andrew J. Maneffa, Adrian B. Harrison, Stewart J. Radford, A. Steve Whitehouse, James H. Clark, Avtar S. Matharu

**Affiliations:** ^1^ Green Chemistry Centre of Excellence, Department of Chemistry University of York Heslington YO10 5DD United Kingdom; ^2^ Department of Biology University of York Heslington YO10 5DD United Kingdom; ^3^ Nestlé Product Technology Centre Nestec York Ltd. Huntington York YO91 1XY UK

**Keywords:** deep eutectic solvents, differential scanning calorimetry, green chemistry, lactones, rheology

## Abstract

Deep eutectic solvents (DES) are one of the most promising green technologies to emerge in recent years given their combination of environmentally friendly credentials and useful functionalities. Considering the continued search for new DES – especially those that exemplify the aforementioned characteristics, we report the preparation of DES based on natural analogues of l‐ascorbic acid for the first time. The onset of eutectic melting occurred at temperatures far below the melting point of the individual components and resulted in the generation of glass forming fluids with glass transition temperatures, viscosities and flow behavior that are comparable to similar systems. This work expands the current array of DES that can be produced using naturally occurring components, which given their potential to be bio‐derived, interesting physicochemical properties (e. g. propensity to supercool and vitrify) and apparent antibacterial nature, may provide utility within a range of applications.

## Graphical abstract

**Vitamin boost**! Unheralded, naturally‐occurring l‐ascorbic acid (Vitamin C) analogues have been used to form novel DES for the first time. Displaying comparable physicochemical properties to similar state‐of‐the‐art systems, it is hypothesized that these DES may have application as cryoprotectants or antibacterials.

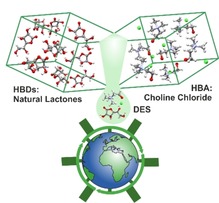

## 1 Introduction

Deep eutectic solvents (DES) are currently one of the most significant areas of research within the wider disciplines of green chemistry and engineering following their introduction as a concept around 15 years ago.^1^, ^2^, ^3^, ^4^ In contemporary literature, the term DES (although ambiguous)^5^ generally describes self‐associating mixtures of two or more Brønsted/Lewis acids and bases which demonstrate a ‘deep’ (i. e. large) melting point depression and thus, a ‘eutectic’ character upon combination, when compared to their native constituents.^6^ Formation of such systems is typically attributed to the establishment of an extensive network of inter‐ and intra‐ molecular interactions, namely hydrogen bonding, between the participating species.^7^ Their ‘green’ credentials (minimal vapor pressure, lack of toxicity, facile preparation, biodegradability etc.)^8^, ^9^ coupled with interesting physicochemical properties (broad polarity range, electrical conductivity, high surface tension, good thermal stability etc.)^4^, ^10^ have prompted the application of DES within a plethora of fields including; organic synthesis, botanicals extraction, biopolymer processing, metal deposition, and biocatalysis.^11^, ^12^, ^13^, ^14^, ^15^

**Figure 1:**
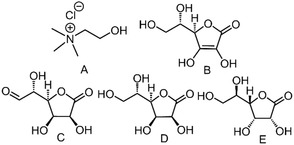
Choline chloride (A) and l‐ascorbic acid (B) analogues; d‐Glucurono‐6,3‐lactone (C, exists as bicyclic structure in crystal), l‐Gulonolactone (D) and d‐Gulonolactone (E).

Due to the breadth of components that can be used to form such media, there are an equal myriad of potential DES and as such, a significant area of research is focused on the exploration of new systems. The search for bio‐based components should be particularly desirable given that most of the current interest surrounding the use of DES is predicated not only on their unusual characteristics, but also environmentally‐friendly attributes ‐ especially when compared to present archetypal fluids (simple molecular solvents, ionic liquids) that they are intending to replace. In this vein, a novel set of DES that was recently highlighted in a patent and subsequent paper involved the combination of choline chloride (ChCl) and the naturally occurring l‐ascorbic acid (Asco, a.k.a. Vitamin C) (Figures 1A and 1B respectively).^16^, ^17^

The Asco:ChCl system has also been shown to be an effective medium in the preparation of so‐called “therapeutic” DES containing dexamethasone for potential utilization within pharmaceutical and/or tissue engineering applications.^18^ The same material has also been trialed as an electrolytic layer in ion‐modulated transistors.^19^ Notably, these examples appear to be some of first DES produced using a lactone constituent, with most instead involving the combination of ChCl and saccharides (e. g. glucose, fructose, sucrose), aliphatic polyols (glycerol, ethylene glycol), carboxylic acids (e. g. lactic acid, malic acid, malonic acid, citric acid) or urea.^20^ Within nature, d‐Glucurono‐6,3‐lactone (GluLac) and l‐Gulonolactone (l‐GLac) (Figures 1C and 1D respectively) are both intermediates in the biosynthesis of Asco.^21^ GluLac is currently utilized as an ingredient in energy drinks,^22^ can be extracted from spruce wood and has also been purported to exist in plant gums,^23^, ^24^ whilst high levels of l‐Glac have been reported for instance, in heat‐stressed soybean seeds.^25^ In light of the aforementioned works concerning the preparation of DES using Asco, it was hypothesized that these structurally similar compounds, in addition to the enantiomer of l‐GLac, d‐Gulonolactone (d‐GLac, Figure 1E), may also be capable of forming DES.

Considering their potential to be derived from natural sources coupled with an anticipated lack of toxicity, the substrates outlined above appear to be excellent new candidates for DES constituents. However, eutectic mixtures formed using these compounds have not been reported previously to the best of our knowledge. Herein, we describe the preparation and characterization of novel DES based on the three aforementioned l‐ascorbic acid analogues. Various physicochemical properties and preliminary bacterial toxicity studies of these new systems are investigated and discussed within the context of similar known DES existing in the literature. Given these attributes, we also highlight potential applications for which they may be well suited.

## 2 Results and Discussion

### 2.1 Formation and Thermal Characteristics of DES

**Figure 2:**
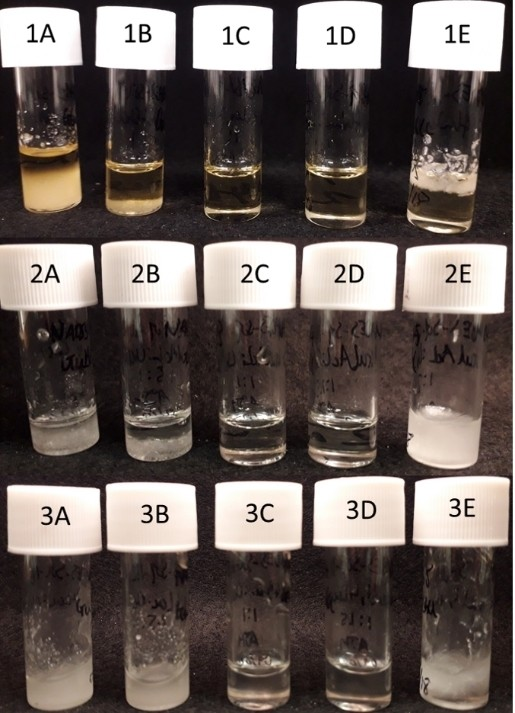
Visual appearance of mixtures comprising either GluLac (1), d‐GLac (2) or l‐GLac (3) and ChCl at varying molar ratios (A: 2 : 1, B: 1.5 : 1, C: 1 : 1, D: 1 : 1.5, E: 1 : 2) following at least 7 days storage at RT.

In this current work, the heating with stirring method was used to screen mixtures of GluLac, l‐GLac or d‐GLac and ChCl at different compositions (2 : 1 to 1 : 2 molar ratio Lactone:ChCl. From visual inspection, only ratios of 1 : 1 and 1 : 1.5 yielded systems whose liquidi readily fell below the operational temperatures (100–110 °C) and also which remained stable liquids (for at least 7 days) at RT (Figure 2). As such, we may infer that these compositions should be the closest to the eutectic composition of the lactone/ChCl mixtures. A similar result was also found for the Asco:ChCl system whereby only ratios of 1 : 1 and 1 : 1.5 were entirely devoid of any crystalline matter under the same storage conditions (Figure S1). Notably, this range is narrower than that reported by Lui et al. (1 : 1.1 to 1 : 2.5)^17^ but in keeping with results for other DES prepared with the most structurally similar hydrogen bond donors (i. e. cyclic polyhydroxy species) given in literature e. g. xylose and glucose which tend to be around 1 : 1 hydrogen bond donor (HBD):ChCl.^26^, ^27^

**Figure 3:**
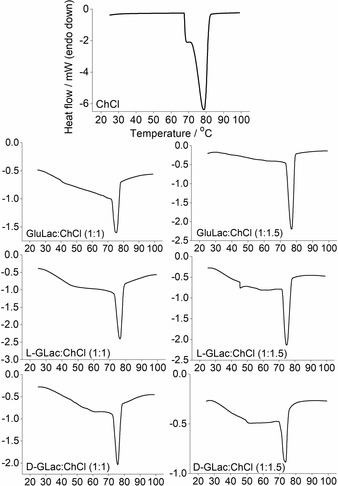
in‐situ DSC traces of the first heat cycles for pure ChCl and lactone‐based DES mixtures.

**Figure 4:**
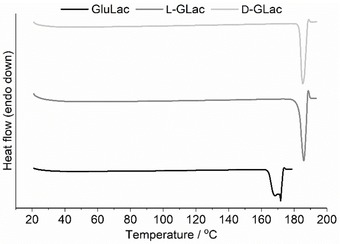
DSC thermograms of pure lactones; GluLac (bottom), l‐GLac (middle) and d‐GLac (top).

The melting point lowering in the eutectic forming systems (Lactone:ChCl, 1 : 1/1 : 1.5) was demonstrated via in‐situ DSC (Figure S2), where endothermic deviations from the baseline can be readily observed starting from ca. 25 °C in all cases (Figure 3). Unsurprisingly, this endothermic behavior was not observed in pure ChCl, with the only visible signal corresponding to the polymorphic transition (at ca. 70–80 °C) from the so‐called α to β form of crystalline ChCl.^26^, ^28^ Interestingly, it appears that the onset of melting (i. e. solidus, also sometimes referred to as the “eutectic temperature”)^29^ in the mixtures occurs at a temperature far below (ΔT_m_>140 °C) that of ChCl (decomposes at ca. 300 °C)^1^ or any of the lactone components, which were found to have melting points of ca. 170, 186, and 185 °C for GluLac, l‐GLac and d‐GLac respectively (Figure 4) (GluLac: lit.,^30^ 176–178 °C. l‐GLac and d‐GLac: lit.,^31^ 186 and 186 °C). According to the general consensus in literature, such melting depression in ChCl‐containing systems is ultimately described as a manifestation of strong hydrogen bonding between it and an added HBD species.^1^, ^2^ As such, we can infer that the lactones used presently must be capable of establishing such interactions when combined with ChCl.

**Figure 5:**
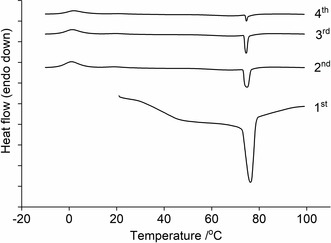
The first, second, third and fourth heat cycles of the in‐situ preparation of l‐GLac:ChCl, 1 : 1. Tick marks along the y‐axis correspond to 0.5 mW.

In all cases, the signal corresponding to ChCl is present in the initial heating cycle although the maxima occur at slightly lower temperatures in the DES mixtures compared to the pure material. This behavior has been described previously for ChCl‐containing systems with various HBD species,^26^ although it was preformed (and not in‐situ prepared) DES containing a molar excess of ChCl (with respect to the eutectic composition) that was examined in the aforementioned study. In the in‐situ experiments, the signals corresponding to ChCl became progressively smaller, suggesting that the mixture was gradually converted to a fluid DES (or at least one which became increasingly devoid of crystalline ChCl). However, formation did not always reach completion, as exemplified in the case of l‐GLac:ChCl, 1 : 1, where ChCl polymorphism could still be detected after the four heat cycles (Figure 5).

**Figure 6:**
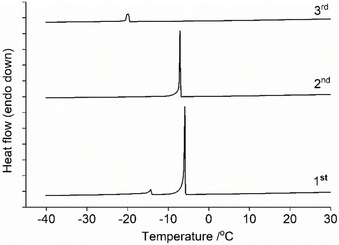
The first, second and third cooling cycles of the in‐situ preparation of l‐GLac:ChCl, 1 : 1. Tick marks along the y‐axis correspond to 1 mW.

Surprisingly, exotherms were also observable during the second, third and fourth heat cycles (i. e. following cooling) at approximately 0 °C. We tentatively hypothesize that these small, but reproducible signals could be the result of a concurrent event whereby the melting of a minimal amount of ice creates sufficient mobility in the amorphous DES matrix for the cold crystallization of a small quantity of the DES component (again most probably ChCl) to occur. The measured water contents of the DES were found to be ca. 0.9 to 1.2 wt. % according to Karl Fisher titration although it should be noted that these values were obtained from analysis of the preformed material and not the solid lactone/ChCl mixtures used for the in‐situ experiments. However, the discrepancies between the various systems are not expected to be significant given that they were all exposed to the same external environment (i. e. ambient air) for comparable amounts of time (ca. 1–2 minutes). Additionally, the possibility of moisture absorption during DSC experimentation was minimized via use of hermetically sealed apparatus. It was also possible to observe several other exothermic signals in all cooling cycles within the region of −20 to −5 °C (Figure 6). Presumably, these are the result of the melt crystallization of either DES constituent or water, as has been described previously for systems comprising ChCl and different HBDs.^27^ The former appears to be more likely given that in some cases, these exotherms actually occur above 0 °C (Figure S2). It is also plausible that they could correspond to the transition of the ChCl β polymorph back to the α form, which is known to occur upon natural cooling (at least for the pure crystal).^32^ In any case, owing to the prominence of the polymorphic signal upon reheating (and the comparative lack of notable melting) we conclude that these exothermic signals most probably originate from ChCl.

It is important to note that the aforementioned thermal events were only evident in the in‐situ experiments where the two DES components were held at 100 °C for 20 minutes per heating cycle as opposed to the 120 continuous minutes (with constant agitation) taken for the experiments conducted using the analogous preformed DES (see Experimental). As such, it is hypothesized that they are most likely the result of limited mixing, which has been described as an important process for “establishing entropy” that is prerequisite for the formation of a DES system.^20^ The lack of agitation could also account for the potential ice formation given that an unmixed matrix would be more likely to exhibit some level of heterogeneity.

**Figure 7:**
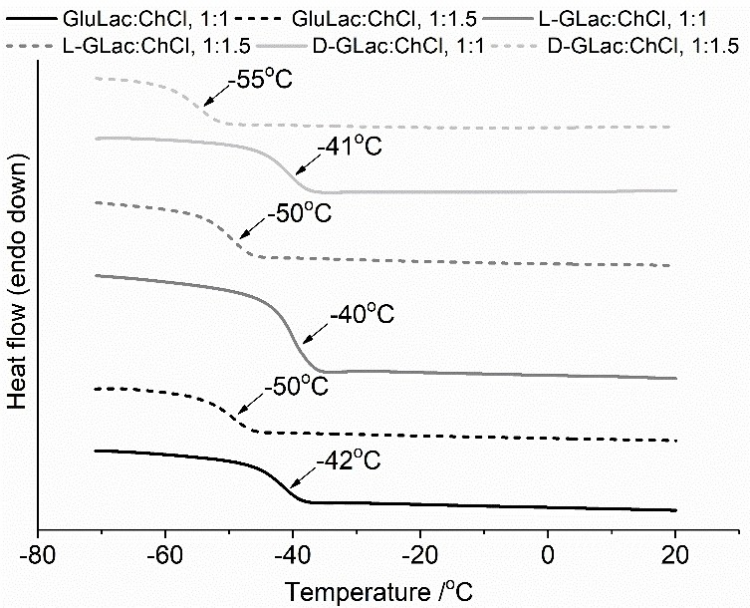
Comparison of the second heat cycles of preformed DES showing the step‐change in heat flow corresponding to the glass transition.

All of the lactone/ChCl mixtures were found to be glass formers, exhibiting a step change in the heat flow which was ascribed to a glass transition, with the midpoint of the discontinuity that occurred during the second heat cycle (Figure 7) taken to be the glass transition temperature (Tg) (full traces of the preformed DES exhibiting distinct glass transitions are shown in Figure S3). The development of glasses within the in‐situ experiments could be observed following initial melting and occurred at similar temperatures with respect to the preformed systems (Figure S4). These results are consistent with previous reports that similar DES (ChCl with sugars) are also glass forming liquids with Tg values that are broadly comparable (ca. −35 to −50 °C) to those found in the present study.^27^, ^33^ The systems containing greater amounts of ChCl (i. e. 1 : 1.5, lactone:ChCl) consistently had a lower Tg when compared with the equimolar mixtures. It should be noted that according to the Karl Fischer analyses, the DES containing more ChCl had marginally higher water contents (possibly due to greater quantity of the highly hygroscopic ChCl). As such, these mixtures may have experienced a slight reduction in Tg given the considerable plasticizing effect of water (only if distributed amongst the main DES components and not involved in the formation of the hypothetical crystalline ice).

In the case of the Asco DES, Karl Fisher titration could not be used for water content determination owing to the unavoidable reduction of iodine by ascorbic acid. As such, it was instead estimated via thermogravimetric analysis ‐ whereby the water content was considered to be equal to the mass loss upon heating to ca. 150 °C (i. e. sufficiently in advance of any loss that could be attributed to thermal decomposition). Using this technique, values of 1.4 and 1.6 wt. % H_2_O were found for the 1 : 1 and 1 : 1.5 DES respectively (Figure S5).

The ability for the DES to supercool far below 0 °C and subsequently undergo vitrification could be beneficial for low‐temperature applications such as cryoprotection, where the amorphous nature and absence of crystallinity in the protective media is advantageous for the preservation of labile substrates. Indeed, such an approach has recently been outlined in the case of other DES systems and appears to offer a promising alternative to current preeminent cryoprotectants such as dimethyl sulfoxide, and ethylene/propylene glycols which have inherent toxicity issues.^34^, ^35^

**Figure 8:**
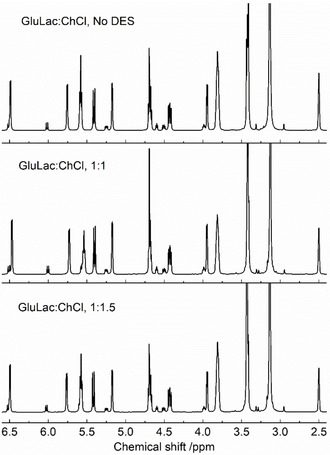
^1^H NMR spectra for a mixed solution of crystalline GluLac and ChCl and diluted GluLac‐containing DES (prepared in DMSO‐d_6_ at ca. 50 mg mL^−1^).

In order to ensure that the initial components had not undergone degradation or reaction during DES preparation, each system was compared with the pure mixed constituents via ^1^H NMR, as exemplified for the GluLac‐containing DES (Figure 8) (Spectra for l‐GLac, d‐GLac and Asco are presented in Figures S6, S7 and S8 respectively). In all cases, spectra of the crystalline lactone and choline chloride were virtually indistinguishable from the corresponding diluted DES systems, with any differences (e. g. relative signal sizes) reflecting the varying ratios of lactone and ChCl that were present in solution (DES concentration was ca. 50 mg mL^−1^ in all cases). The omnipresent peak at 2.50 ppm corresponds to the solvent (DMSO) whilst the signal at ca. 3.38 ppm can be attributed to water, which appears to overlap with that belonging to the methylene group neighboring the nitrogen in ChCl. The complete conversion of starting materials into the DES is particularly notable as it ensures that their production is 100 % atom economical and mitigates the generation of undesirable by‐products which have occasionally been reported when using conventional HBDs such as carboxylic acids.^36^, ^37^

The preservation of starting constituents could also be important when considering the potential safety of the lactone‐DES. GluLac is an established beverage ingredient which can be present at the gram scale within consumer products.^38^ Similarly, choline chloride has also been listed as an ingredient in infant formulas and appears to be considered a safe substance by the Food and Drug Administration.^39^, ^40^

### 2.2 Bacterial Toxicity Testing

**Figure 9:**
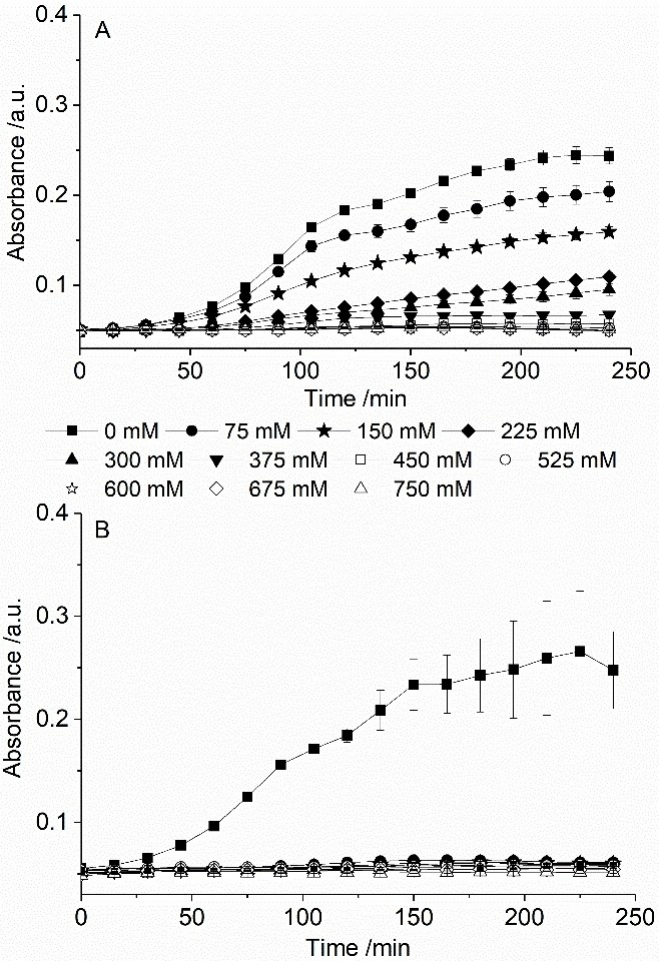
Evolution of optical density (620 nm) for LB media containing l‐Glac:ChCl, 1 : 1 (A) and Asco:ChCl, 1 : 1 (B) at 0 to 750 mM. Error bars represent SE.

Given the aforementioned safety credentials of the starting materials, we anticipated that DES formed using these constituents should constitute benign media for most applications and even those involving direct human usage. However, given the ambivalence of previous investigations, a preliminary toxicity study of the novel DES systems was undertaken.^41^ Based on methodology outlined in relevant recent literature, this involved tracking the growth (or retardation thereof) of Escherichia coli (E. coli, strain BL21) within a Luria‐Bertani medium in the presence of either Asco or lactone‐DES (75–750 mM) via optical density measurements of at 620 nm (Figure 9).^42^

For lactone‐based DES, bacterial growth rate was found to be inversely correlated with concentration (as exemplified for the l‐GLac:ChCl, 1 : 1 system (Figure 9A)) and independent of HBD identity or molar composition (Figure S9). In contrast, both DES formed using l‐ascorbic acid appeared to completely inhibit E. coli growth at any non‐zero concentration (1 : 1 mixture is shown in Figure 9B) whereas full inhibition was only realized at 375–450 mM (Figure S10) in the presence of a lactone‐DES. The prevention of growth within the systems comprising Asco is expected given the apparent antimicrobial action of the native constituent.^43^ It is possible that this inhibition is predominantly a result of pH lowering of the growth media as has been suggested for other studies involving acidic HBDs.^44^ We anticipate that these potential antimicrobial properties may augment the utility of so‐called “therapeutic” DES based on Asco within applications that require microbiologically sterile conditions.

### 2.3 Rheological Behavior

**Figure 10:**
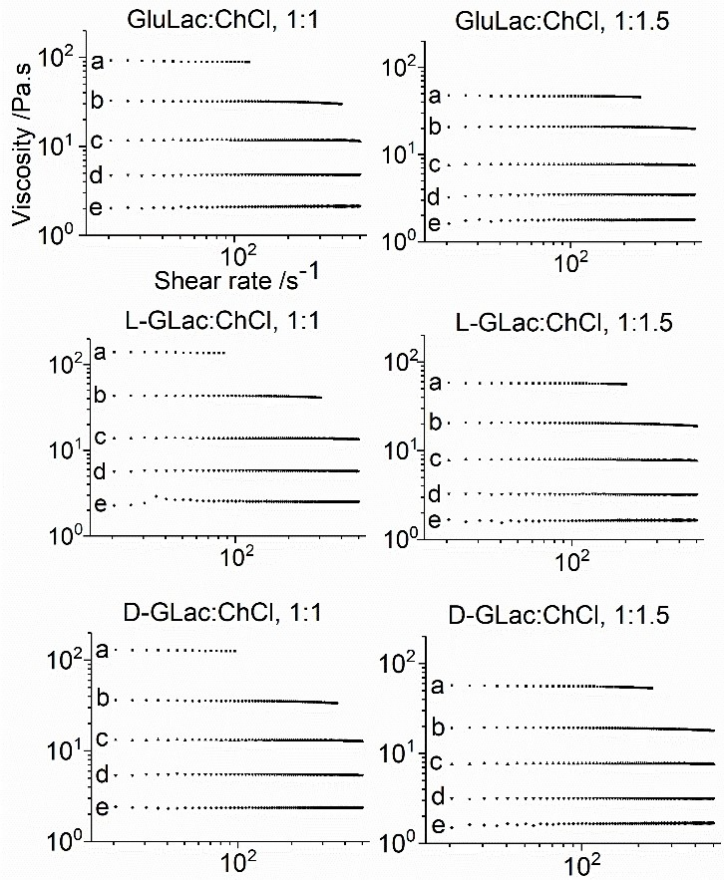
Viscosity (in Pa.s) as a function of shear rate (s^−1^) at *T*=20 to 60 °C (a to e) for each lactone‐DES.

All of the six lactone‐based DES were found to be viscous fluids that displayed Newtonian flow behavior at low to moderate shear rates (<500 s^−1^) (Figure 10). It should be noted that our displayed data starts from 20 s^−1^ as below this value, there was often little repeatability across multiple replicates suggesting they were potentially experimental artefacts (full traces are provided in Figure S11). It is also pertinent to recognize that on account of the large shear rate gradient, seemingly erroneous measurements essentially corresponded to single data points that were not reproducible. Hence, we emphasize that our goal is to highlight the qualitative, reproducible trends rather than individual quantitative datum. Similar Newtonian behavior has been reported previously in various other sugar (glucose, xylose) or sugar alcohol (xylitol, sorbitol) containing DES.^26^, ^33^, ^45^

The measured viscosity for all of the novel DES were found to be very comparable to the Asco:ChCl system which varied between approximately 8 to 250 and 2 to 85 Pa.s (mean averages) for the 1 : 1 and 1 : 1.5 mixtures (at 20 to 60 °C) respectively. Interestingly, we note that our values are significantly higher when compared to the Asco:ChCl system as reported by Liu et al., although those authors provided minimal details concerning the nature of the rheological experiments and initial water contents of the examined DES.^17^ The results are closer to the values suggested for Asco:ChCl, 1 : 2 by Silva et al. albeit with a different measuring geometry.^18^ However, again water content was not documented and it is unclear whether any precautions were taken to prevent moisture absorption during experimentation. As expected, the viscosity was found to be inversely related to temperature, as has been documented in other DES systems for instance Xylose:ChCl, 1 : 1, which Aroso et al. reported to have a viscosity of ca. 90, 25, 9, 4 and 2 Pa.s at 20, 30, 40, 50, 60 °C respectively with a water content of 1 wt. %.^26^

**Figure 11:**
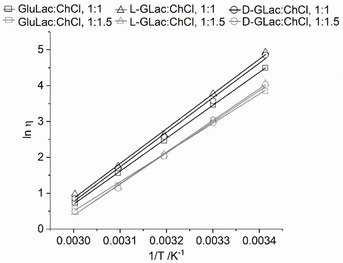
Arrhenius plots for each lactone‐DES at *T*=20, 30, 40, 50 and 60 °C (at a shear rate of 50 s^−1^).

The viscosity/temperature relationship can be well described using an Arrhenius model (Equation (1)

(1) $$\eta =\eta_{0}\;e^{\left(\frac{E_{a}}{RT}\right)}$$

where *η* corresponds to the viscosity (Pa.s), *η_0_* is a pre‐exponential constant, *Ea* is the activation energy (kJ mol^−1^), *R* is the universal gas constant (8.314 J K^−1^ mol^−1^) and *T* the absolute temperature (K). All six of the lactone‐DES displayed linear plots for Equation 1 (Figure 11) with excellent fits (R^2^>0.997) and model parameters (Table 1) comparable to those quoted in literature (results of this work were obtained using the viscosity measured at a shear rate of 50 s^−1^).^26^, ^46^ This was also found to be the true for the Asco‐containing systems (Figure S12, Table S2). It appears as though the most discriminating factor for the value of viscosity was the lactone:ChCl molar ratio, with the identity of the HBD having comparatively little influence. The l‐GLac and d‐GLac systems behaved almost identically which is not surprising given their close molecular resemblance.

**Table 1:** Comparison of the parameters for the Arrhenius equation (*η* ^*0*^, *Ea* and R^2^) of lactone‐DES and selected values from literature.

System, molar ratio	*η*_0_ [Pa.s]	*Ea* [kJ mol^−1^]	R^2^
GluLac:ChCl, 1 : 1^[a]^	2.057 E‐12	76.495	1.000
GluLac:ChCl, 1 : 1.5^[a]^	3.742 E‐11	67.920	0.998
l‐GLac:ChCl, 1 : 1^[a]^	5.295 E‐13	80.708	0.997
l‐GLac:ChCl, 1 : 1.5^[a]^	4.521 E‐12	73.447	0.999
d‐GLac:ChCl, 1 : 1^[a]^	5.787 E‐13	80.220	0.997
d‐GLac:ChCl, 1 : 1.5^[a]^	6.927 E‐12	72.270	0.998
d‐Glucose:ChCl, 1 : 1.5^[b]^	5.772 E‐13	74.991	–
d‐Xylose:ChCl, 1 : 1^[c]^	3.627 E‐11	68.530	0.991

[a] This work. [b] From Ref. [46] Water content not disclosed. [c] From Ref. [26] Contained 1 wt. % water.

The viscosity appeared to be consistently lower for the DES containing larger amounts of ChCl when compared to the analogous equimolar systems. Historically, ‘hole theory’ has been used to rationalize the high viscosity of ChCl‐based DES wherein, it is considered a manifestation of the disparity between the large radii of the constituents and the small average void size existing within the liquid matrix.^2^, ^47^ In the present systems, the larger size of the lactone (with complexed chloride anion) compared to the choline cation should result in a lower probability of locating suitably sized holes and hence, a higher viscosity. This is reflected in the activation energies, which are all consistently smaller in the case of the DES prepared from the 1 : 1.5 lactone:ChCl mixtures. However, the applicability of hole theory should be investigated further given recent suggestions that void dimensions within DES may be considerably smaller than previously thought.^48^ Interestingly, an inverse relationship between viscosity and ChCl concentration has also been reported previously in glycerol:ChCl DES.^49^, ^50^ This result was attributed to the ability of the salt to interfere with the three dimensional hydrogen‐bond network by partially complexing the glycerol hydroxyls with Cl^−^. A similar effect may also be plausible in the case of the present lactone‐based systems given that the large number and broad spatial distribution of hydroxyl groups on the lactone would presumably result in extensive and multi‐directional hydrogen bonding throughout the fluid.

The high viscosity of the present DES under ambient conditions may situationally limit their utilization in certain applications, although it should be less problematic at sufficiently elevated temperatures in accordance to the Arrhenius relation as described above. Inspection of the ^1^H NMR results (Figures 8, S6 and S7) suggests that the lactone‐DES appear to stable against thermal degradation at temperatures which should suffice for most applications (100/110 °C). Besides adjusting temperature, it may also be possible to modify the rheological properties via the addition of small amounts of a third, low viscosity component (e. g. a molecular liquid).

## 3 Conclusions

The present study outlines the preparation of new DES using choline chloride and three novel l‐ascorbic acid analogues. All mixtures displayed eutectic melting at temperatures considerably lower than the melting points of the individual constituents (*ΔT_m_*>140 °C) with the eutectic compositions appearing to lie around 1 : 1–1 : 1.5 lactone:ChCl molar ratio according to visual evaluation. The resulting melts were all found to be viscous, glass forming fluids with properties (glass transition temperatures and viscosities) comparable to previous DES comprising the most structurally similar HBDs. Preliminary bacterial testing indicated that the DES had a moderate inhibitory effect on the proliferation of E. Coli. when compared to existing mixtures based on l‐ascorbic acid.

The results of this study broaden the current pool of potential bio‐based DES components and appear to be some of the first examples where naturally‐occurring lactones are employed as hydrogen bond donor species. Given the preservation and seemingly innocuous nature of the constituents involved, the new DES highlighted herein present attractive alternatives for several pre‐existing eutectics and may find application within cryoprotection or microbiological treatment.

## Experimental Section

### Materials

Asco (99 %, CAS: 50‐81‐7), ChCl (>98 %, CAS: 67‐48‐1), GluLac (>99 %, CAS: 32449‐92‐6) and l‐GLac (95 %, CAS: 1128‐23‐0), DMSO‐d_6_ (99.9 atom % D) and silicone oil (5 cSt at 25 °C) were purchased from Sigma Aldrich. d‐GLac (98 %, CAS: 6322‐07‐2) was purchased from Alfa Aesar. Hydranal Composite 5 K and Hydranal KetoSolver were purchased from Thermo Fisher Scientific. All chemicals were used as received and all DES were stored in a desiccator following preparation.

### Deep Eutectic Solvent Preparation

DES mixtures were prepared by weighting desired amounts of HBD (Asco, GluLac, l‐GLac or d‐GLac) with ChCl to give molar ratios of 1 : 2, 1 : 1.5, 1 : 1, 1.5 : 1 and 2 : 1. These solid mixtures were placed into sealed glass vials and stirred at a constant 100 °C (Asco, GluLac) or 110 °C (l‐GLac, d‐GLac) for 2 hours, before being allowed to naturally cool to ambient temperature.

### Differential Scanning Calorimetry (DSC)

All DSC measurements were performed using a TA Instruments Q2000 DSC under a nitrogen atmosphere and hermetically sealed aluminium pans containing ca. 6–10 mg of sample. A heating rate of 3 °C min^−1^ was used for the melting point determination of the pure lactones (temperatures ranges of 20 to 180 (GluLac) or 20 to 195 °C (l‐/d‐GLac) and during the heating cycles of both the in‐situ preparation and Tg determination (using preformed material) of DES for which, the full procedure is outlined in Table S1. The Tg was calculated from the step‐change in heat flow that occurred during the second heating cycle using the ‘Tg analysis’ tool available within the Universal Analysis 2000 software (TA Instruments).

### Rheological Measurements

All rheological experiments were conducted using a Brookfield CPS+ Rheometer fitted with cone/plate geometry (Cone: diameter=25 mm, angle=2°, gap height=0.045 mm) and a Peltier PTS‐2 temperature controller at atmospheric pressure. A fresh aliquot of DES (ca. 0.3 mL) was applied to the bottom measuring plate at 20 °C before both silicone oil and a solvent trap were placed around it in order to limit moisture absorption. The methodology used in the present study was based on a modification of a previous description in the literature.^51^ Briefly, the sample was heated to and equilibrated at the desired temperature (20, 30, 40, 50 or 60 °C) for 10 minutes before being subjected to a 120 s pre‐shear at 20 Pa followed by a 60 s equilibration to remove rheological history. The rheological properties of each DES were investigated using a controlled shear rate regime whereby the shear rate was increased linearly from 5 to 500 s^−1^ over a period of 100 s (i. e. a 5 s^−1^ increase per second). Each reported result reflects the average of at least two replicates.

### Water Content Analysis

Water content analyses were carried out via volumetric Karl Fisher titration using a 907 Titrando unit (Metrohm) whereby Hydranal Composite 5 K and Hydranal KetoSolver were used as the titrant and working medium respectively. A polarizing current of 50 μA, stop voltage of 250 mV and a drift end‐point criterion (20 μL min^−1^) was applied in all cases. All titrations were carried out ca. 22 °C with a minimal duration of 120 s and sample sizes of approximately 1–1.5 g. Each titration was conducted in triplicate.

### ^1^H NMR Spectroscopy

All ^1^H NMR spectroscopy was performed using a JEOL JNM‐ECS400 A spectrometer operating at a frequency of 400 MHz and a temperature of ca. 25 °C. Samples were prepared by dissolving either the preformed DES or crystalline components (in an approximately 1 : 1.5, lactone:ChCl molar ratio) in DMSO‐d_6_ to give solutions of ca. 50 mg mL^−1^.

### Thermogravimetric Analysis

Thermogravimetric analysis of l‐ascorbic acid DES was conducted using a PL Thermal Sciences STA 625 in which samples (ca. 10 mg) were heated in aluminium cups under a flow of nitrogen (ca. 50 mL min^−1^) to 625 °C at a rate of 10 °C min^−1^.

### Bacterial Toxicity Testing

Samples of the DES (either 1 M or 0.83 M) were dissolved in Luria‐Bertani medium (LB) before being filter sterilized through a 0.45 μm Filtropur syringe filter (Starstedt) to remove any contaminating bacteria. An overnight culture of E. coli BL21 (DE3) (Novagen) was grown in LB at 37 °C. From this, 1.0 ml was taken and sterile LB added until the absorbance at 620 nm of this sample was 0.4. Using a previously prepared calibration curve this corresponds to approximately 4×10^8^ bacteria per ml.

Growth experiments were conducted by mixing the bacterial suspension, test samples and sterile broth in the correct ratios to achieve 100 μl samples with the desired concentration of the compound and a constant number of bacteria (approximately 1×10^7^). Experiments were performed in sterile 96 well clear flat bottom polystyrene culture plates (Corning). Samples were incubated at 37 °C for 4 h and growth determined by measuring absorbance at 620 nm every 15 minutes using a Multiskan FC Microplate Photometer (ThermoFisher Scientific). All concentrations were tested in triplicate in the range 0 (control) to 750 mM at 75 mM intervals.

## Conflict of interest

The authors declare no conflict of interest.

## Supporting information

As a service to our authors and readers, this journal provides supporting information supplied by the authors. Such materials are peer reviewed and may be re‐organized for online delivery, but are not copy‐edited or typeset. Technical support issues arising from supporting information (other than missing files) should be addressed to the authors.

Supplementary

## References

[1] A. P. Abbott, G. Capper, D. L. Davies, R. K. Rasheed, V. Tambyrajah, *Chem. Commun* **2003**, 70. 10.1039/b210714g 12610970

[2] A. P. Abbott , D. Boothby , G. Capper , D. L. Davies , R. K. Rasheed , J. Am. Chem. Soc. 2004, 126, 9142. 15264850 10.1021/ja048266j

[3] C. J. Clarke , W. C. Tu , O. Levers , A. Bröhl , J. P. Hallett , Chem. Rev. 2018, 118, 747. 29300087 10.1021/acs.chemrev.7b00571

[4] I. Wazeer , M. Hayyan , M. K. Hadj-Kali , J. Chem. Technol. Biotechnol. 2018, 93, 945.

[5] E. Durand , J. Lecomte , P. Villeneuve , Biochimie 2016, 120, 119. 26391220 10.1016/j.biochi.2015.09.019

[6] A. Paiva , R. Craveiro , I. Aroso , M. Martins , R. L. Reis , A. R. C. Duarte , ACS Sustainable Chem. Eng. 2014, 2, 1063.

[7] L. I. N. Tome , V. Baiao , W. da Silva , C. M. A. Brett , Appl. Mater. Today 2018, 10, 30.

[8] Q. Zhang , K. De Oliveira Vigier , S. Royer , F. Jerome , Chem. Soc. Rev. 2012, 41, 7108. 22806597 10.1039/c2cs35178a

[9] E. L. Smith , A. P. Abbott , K. S. Ryder , Chem. Rev. 2014, 114, 11060. 25300631 10.1021/cr300162p

[10] G. Garcia , S. Aparicio , R. Ullah , M. Atilhan , Energy Fuels 2015, 29, 2616.

[11] S. Khandelwal , Y. K. Tailor , M. Kumar , J. Mol. Liq. 2016, 215, 345.

[12] M. H. Zainal-Abidin , M. Hayyan , A. Hayyan , N. S. Jayakumar , Anal. Chim. Acta 2017, 979, 1. 28599704 10.1016/j.aca.2017.05.012

[13] X. Tang , M. Zuo , Z. Li , H. Liu , C. Xiong , X. Zeng , Y. Sun , L. Hu , S. Liu , T. Lei , L. Lin , ChemSusChem 2017, 1 0, 2696. 10.1002/cssc.201700457 28425225

[14] E. L. Smith , Trans. IMF 2013, 91, 241.

[15] P. Xu , G. W. Zheng , M. H. Zong , N. Li , W. Y. Lou , Bioresour. Bioprocess. 2017, 4, 34. 28794956 10.1186/s40643-017-0165-5 PMC5522511

[16] W. Liu, F. Hou, J. Chen, G. Yang, B. Zong, F. Wang, (Univ. Henan Technology), CN106397082 A, **2016**.

[17] W. Liu , K. Zhang , J. Chen , J. Yu , J. Mol. Liq. 2018, 260, 173.

[18] J. M. Silva , R. L. Reis , A. Paiva , A. R. C. Duarte , ACS Sustainable Chem. Eng. 2018, 6, 10355.

[19] F. Pettersson , T. Remonen , D. Adekanye , Y. Zhang , C.-E. Wilén , R. Österbacka , ChemPhysChem 2015, 16, 1286. 25694168 10.1002/cphc.201402701

[20] Y. Liu , J. B. Friesen , J. B. McAlpine , D. C. Lankin , S.-N. Chen , G. F. Pauli , J. Nat. Prod. 2018, 81, 679. 29513526 10.1021/acs.jnatprod.7b00945 PMC5913660

[21] M. W. Davey , M. Van Montagu , D. Inzé , M. Sanmartin , A. Kanellis , N. Smirnoff , I. J. J. Benzie , J. J. Strain , D. Favell , J. Fletcher , J. Sci. Food Agric. 2000, 80, 825.

[22] T. M. McLellan , H. R. Lieberman , Nutr. Rev. 2012, 70, 730. 23206286 10.1111/j.1753-4887.2012.00525.x

[23] D. Finnegan , BNF Nutr. Bull. 2003, 28, 147.

[24] A. R. N. Gorrod , J. K. N. Jones , J. Chem. Soc. 1954, 2522.

[25] K. K. Chebrolu , F. B. Fritschi , S. Ye , H. B. Krishnan , J. R. Smith , J. D. Gillman , Metabolomics 2016, 12 : 28.

[26] I. M. Aroso , A. Paiva , R. L. Reis , A. R. C. Duarte , J. Mol. Liq. 2017, 241, 654.

[27] R. Craveiro , I. Aroso , V. Flammia , T. Carvalho , M. T. Viciosa , M. Dionísio , S. Barreiros , R. L. Reis , A. R. C. Duarte , A. Paiva , J. Mol. Liq. 2016, 215, 534.

[28] V. Petrouleas , R. M. Lemmon , A. Christensen , J. Chem. Phys. 1978, 68, 2243.

[29] L. J. B. M. Kollau , M. Vis , A. van den Bruinhorst , A. C. C. Esteves , R. Tuinier , Chem. Commun. 2018, 54, 13351. 10.1039/c8cc05815f 30417900

[30] M. Stacey , J. Chem. Soc. 1939, 1529.

[31] H. Flores , P. Amador , J. Chem. Thermodyn. 2004, 36, 1019.

[32] A. Nath , R. Agarwal , R. M. Lemmon , J. Chem. Phys. 1974, 61, 1542.

[33] V. Fischer , W. Kunz , Mol. Phys. 2014, 112, 1241.

[34] V. I. B. Castro , R. Craveiro , J. M. Silva , R. L. Reis , A. Paiva , A. R. C. Duarte , Cryobiology 2018, 83, 15. 29944855 10.1016/j.cryobiol.2018.06.010

[35] B. P. Best , Rejuvenation Res. 2015, 18, 422. 25826677 10.1089/rej.2014.1656 PMC4620521

[36] C. Florindo , F. S. Oliveira , L. P. N. Rebelo , A. M. Fernandes , I. M. Marrucho , ACS Sustainable Chem. Eng. 2014, 2, 2416.

[37] N. R. Rodriguez , A. van den Bruinhorst , L. J. B. M. Kollau , M. C. Kroon , K. Binnemans , ACS Sustainable Chem. Eng. 2019, 7, 11521.

[38] J. Rotstein , J. Barber , C. Strowbridge , S. Hayward , R. Huang , S. B. Godefroy , Int. Food Risk Anal. J. 2013, 3, 4 : 2013.

[39] C.-S. Lai, M. Kuchan, (Abbott Lab), US2016008318A1, **2018**.

[40] A. A. C. T. Hijo , G. J. Maximo , M. C. Costa , E. A. C. Batista , A. J. A. Meirelles , ACS Sustainable Chem. Eng. 2016, 4, 5347.

[41] Y. P. Mbous , M. Hayyan , W. F. Wong , C. Y. Looi , M. A. Hashim , Sci. Rep. 2017, 7, 41257. 28145498 10.1038/srep41257 PMC5286504

[42] X. Marset , J. Torregrosa-Crespo , R. M. Martinez-Espinosa , G. Guillena , D. J. Ramón , Green Chem. 2019, 21, 4127.

[43] R. J. Verghese , S. K. Matthew , A. David , J. Curr. Res. Sci. Med. 2017, 3, 88.

[44] A. Mišan, J. Nađpal, A. Stupar, M. Pojić, A. Mandić, R. Verpoorte, Y. H. Choi, *Crit. Rev. Food Sci. Nutr* **2019**, DOI: 10.1080/10408398.2019.1650717. 10.1080/10408398.2019.1650717 31407921

[45] Z. Maugeri , P. D. de Maria , RSC Adv. 2012, 2, 421.

[46] A. Hayyan , F. S. Mjalli , I. M. AlNashef , Y. M. Al-Wahaibi , T. Al-Wahaibi , M. A. Hashim , J. Mol. Liq. 2013, 178, 137.

[47] A. P. Abbott , G. Capper , S. Gray , ChemPhysChem 2006, 7, 803. 16596609 10.1002/cphc.200500489

[48] O. S. Hammond , D. T. Bowron , K. J. Edler , Green Chem. 2016, 18, 2736.

[49] A. P. Abbott , R. C. Harris , K. S. Ryder , C. D′Agostino , L. F. Gladden , M. D. Mantle , Green Chem. 2011, 13, 82.

[50] A. P. Abbott , R. C. Harris , K. S. Ryder , J. Phys. Chem. B 2007, 111, 4910. 17388488 10.1021/jp0671998

[51] G. L. Burrell , N. F. Dunlop , F. Separovic , Soft Matter 2010, 6, 2080.

