# Supplementary material for: Deep Eutectic Solvents Based on Natural Ascorbic Acid Analogues and Choline Chloride
Source: ChemistryOpen. 2020 May 4;9(5):550–8. doi: 10.1002/open.202000020 (PMC7197082; doi:10.1002/open.202000020)
Supplement: Supplementary file 1 — Supplementary [file OPEN-9-550-s001.pdf]

ChemistryOpen

Supporting Information

Deep Eutectic Solvents Based on Natural Ascorbic Acid Analogues and Choline Chloride

Andrew J. Maneffa, Adrian B. Harrison, Stewart J. Radford, A. Steve Whitehouse, James H. Clark, and Avtar S. Matharu*© 2020 The Authors. Published by Wiley-VCH Verlag GmbH & Co. KGaA. This is an open access article under the terms of the Creative Commons Attribution License, which permits use, distribution and reproduction in any medium, provided the original work is properly cited.Supporting information for this article contains full DSC procedure and heat/cool cycles, ^{1}H NMR spectra, complete rheological traces and comparative experiments using L-ascorbic acid:ChCl DES.

Table S1. Tabulated DSC procedure used to investigate the thermal properties of in-situ and pre-prepared DES.

Step No.	
1	Heat from 20 to 100 ºC at 3 ºC min^{-1}
2	Isothermal at 100 ºC (20 min)
3	Cool from 100 to -80 ºC at 10 ºC min^{-1}
4	Isothermal at -80 ºC (5 min)
5	Heat from -80 to 100 ºC at 3 ºC min^{-1}
	Twice repeat Steps 2 to 5

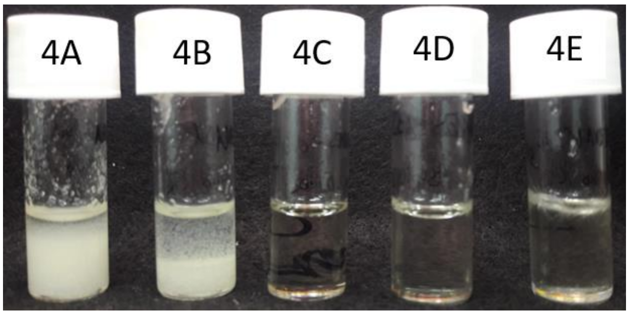

Figure S1. Visual appearance of L-ascorbic acid:ChCl DES (4A, B, C, D and E correspond to molar ratios of 2:1, 1.5:1, 1:1, 1:1.5 and 1:2 respectively).

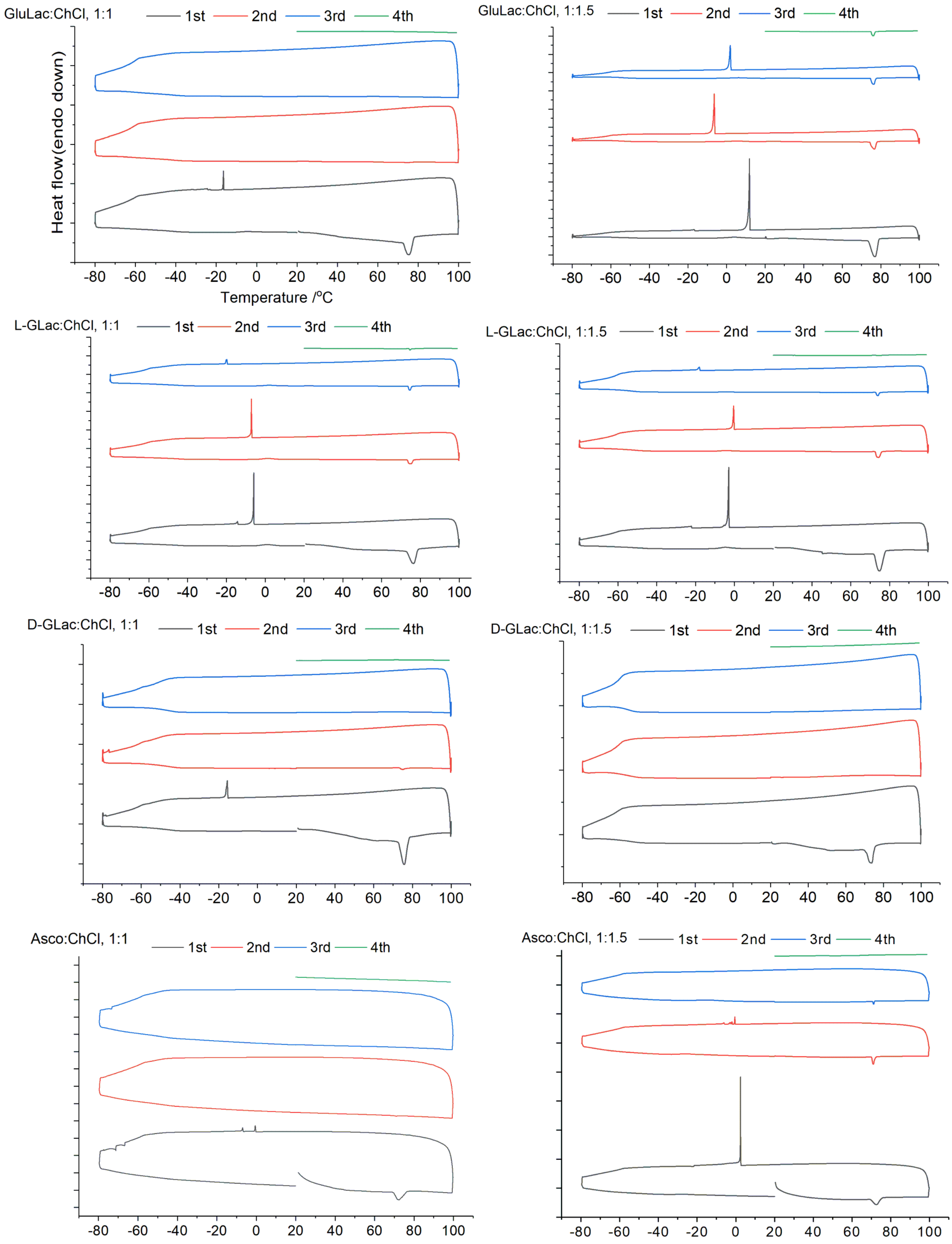

Figure S2. Full DSC traces of in-situ prepared lactone-DES showing the 1st, 2nd and 3rd Heat/Cool and 4th Heat cycles (large y-axis tick marks correspond to 2 mW except for Asco:ChCl, 1:1.5 wherein they correspond to 5mW).

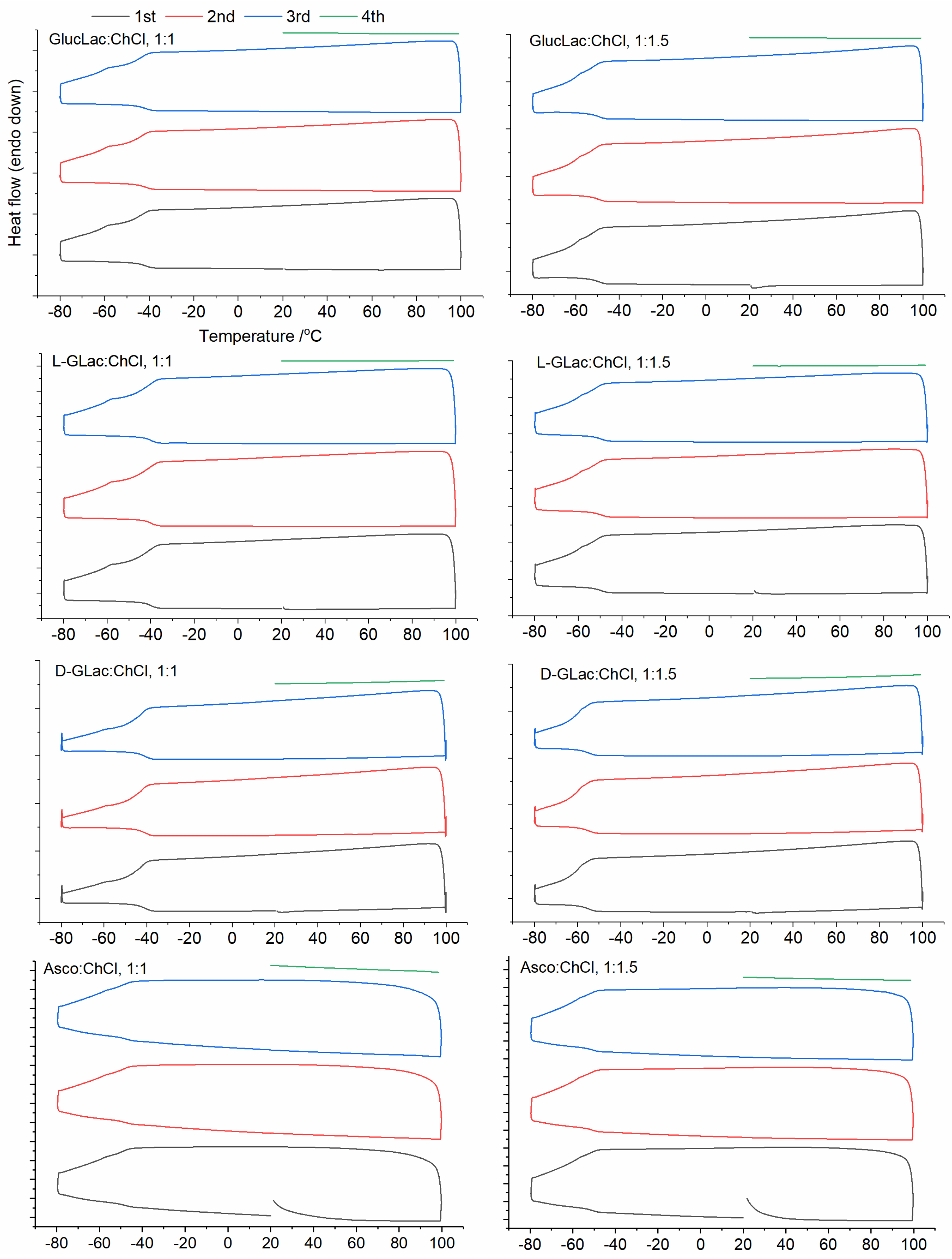

Figure S3. Full DSC traces of preformed lactone- and Asco-containing DES showing the 1st, 2nd and 3rd Heat/Cool and 4th Heat cycles (large y-axis tick marks correspond to 2 mW).

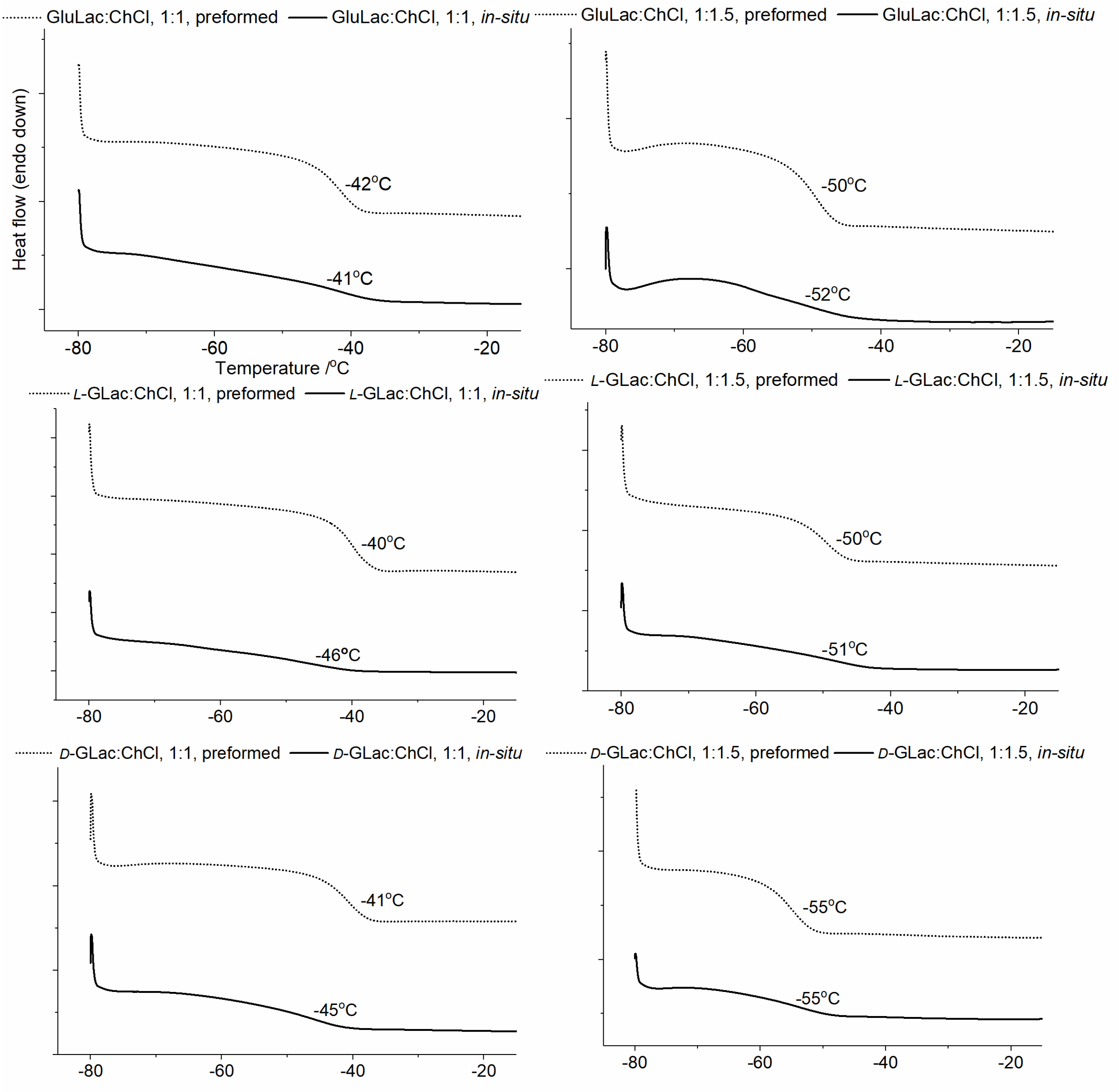

Figure S4. Comparison of glass transition temperatures for in-situ and preformed lactone-DES. Large y-axis tick marks correspond to 0.5 mW in all cases.

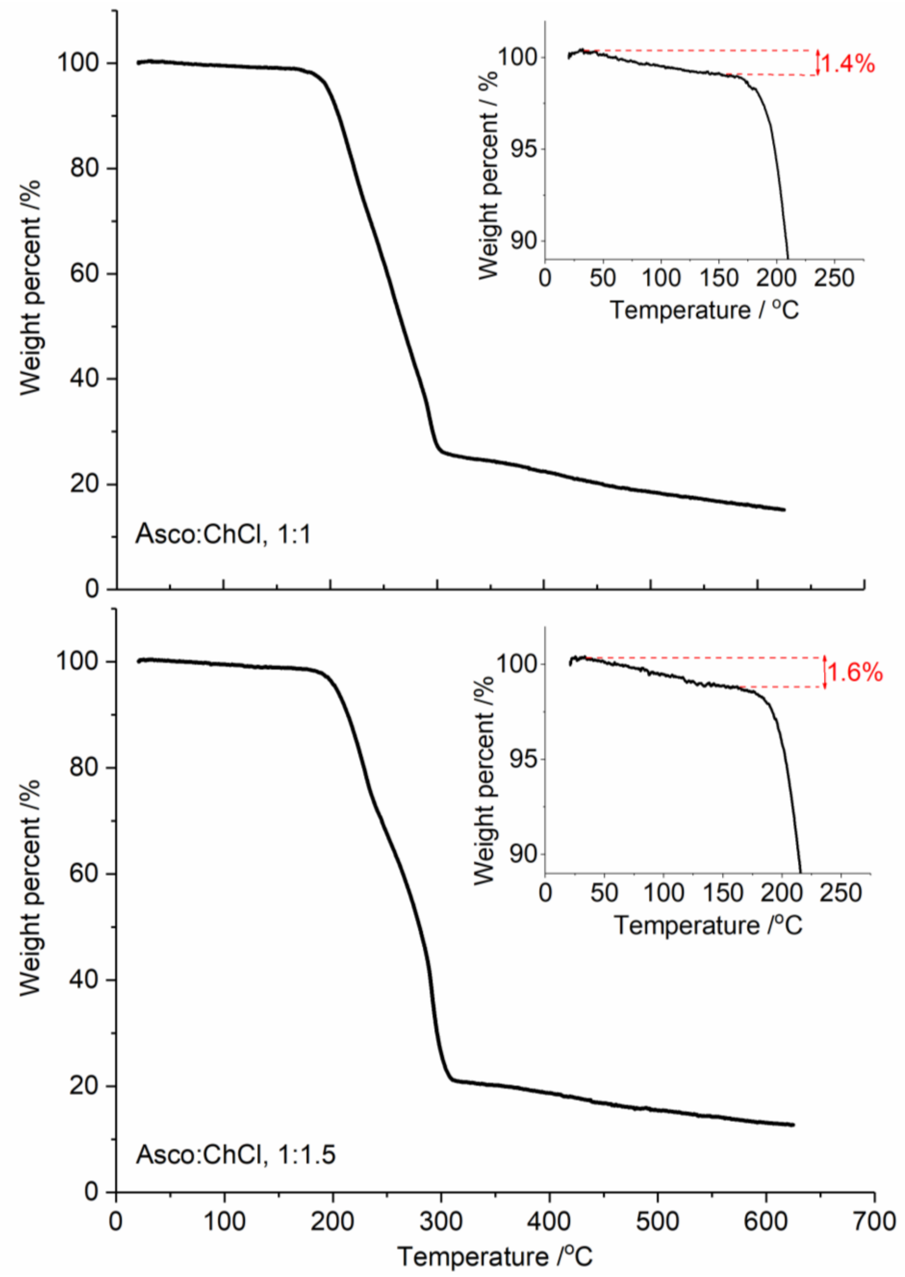

Figure S5. Thermogravimetric analysis of L-ascorbic acid-based DES wherein the initial mass loss is highlighted within the inset.

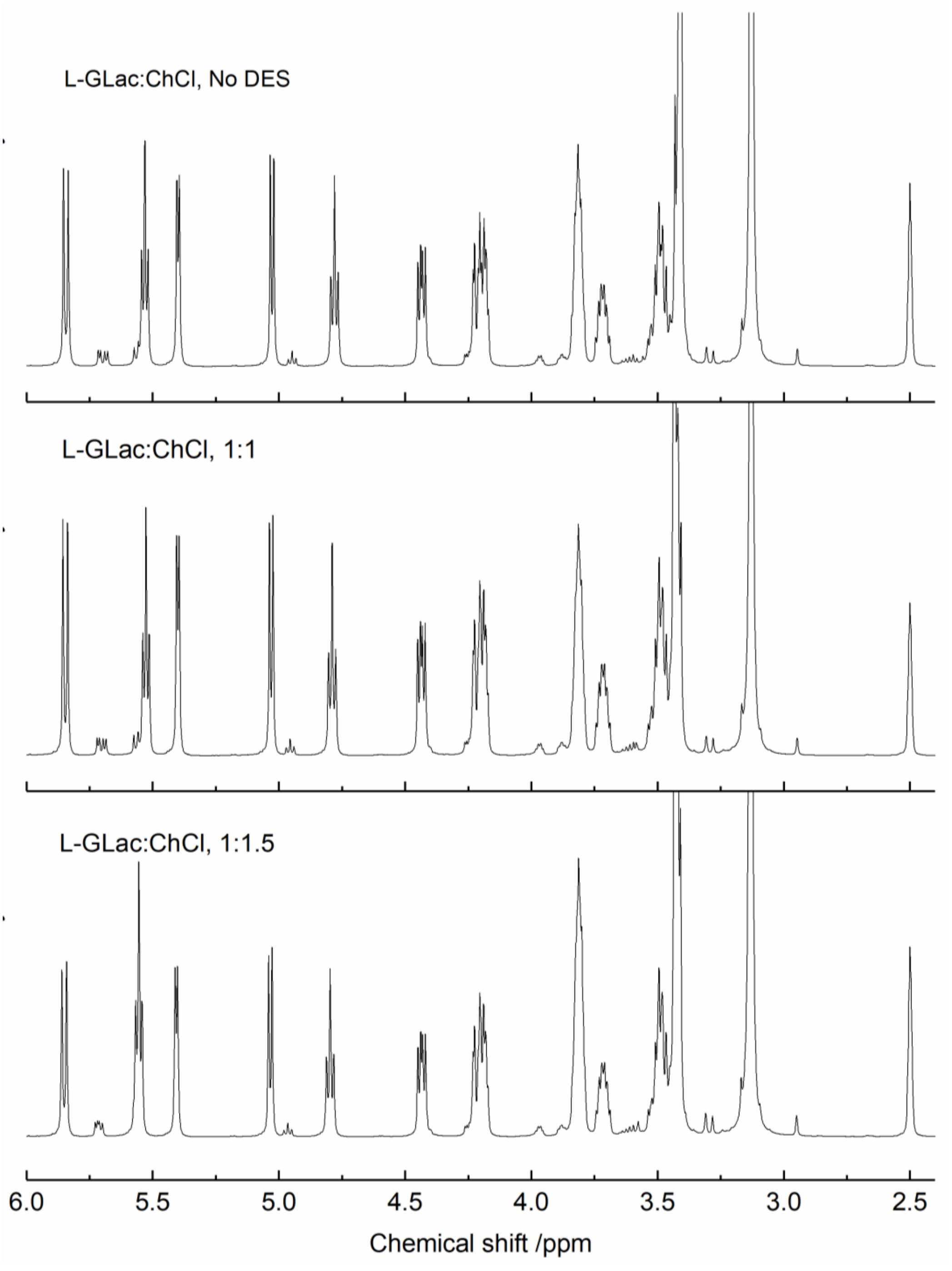

Figure S6. ^1H NMR spectra for a mixed solution of L-GLac and ChCl and diluted L-GLac-containing DES (all prepared in DMSO-d_6, ca. 50 mg mL^{-1}).

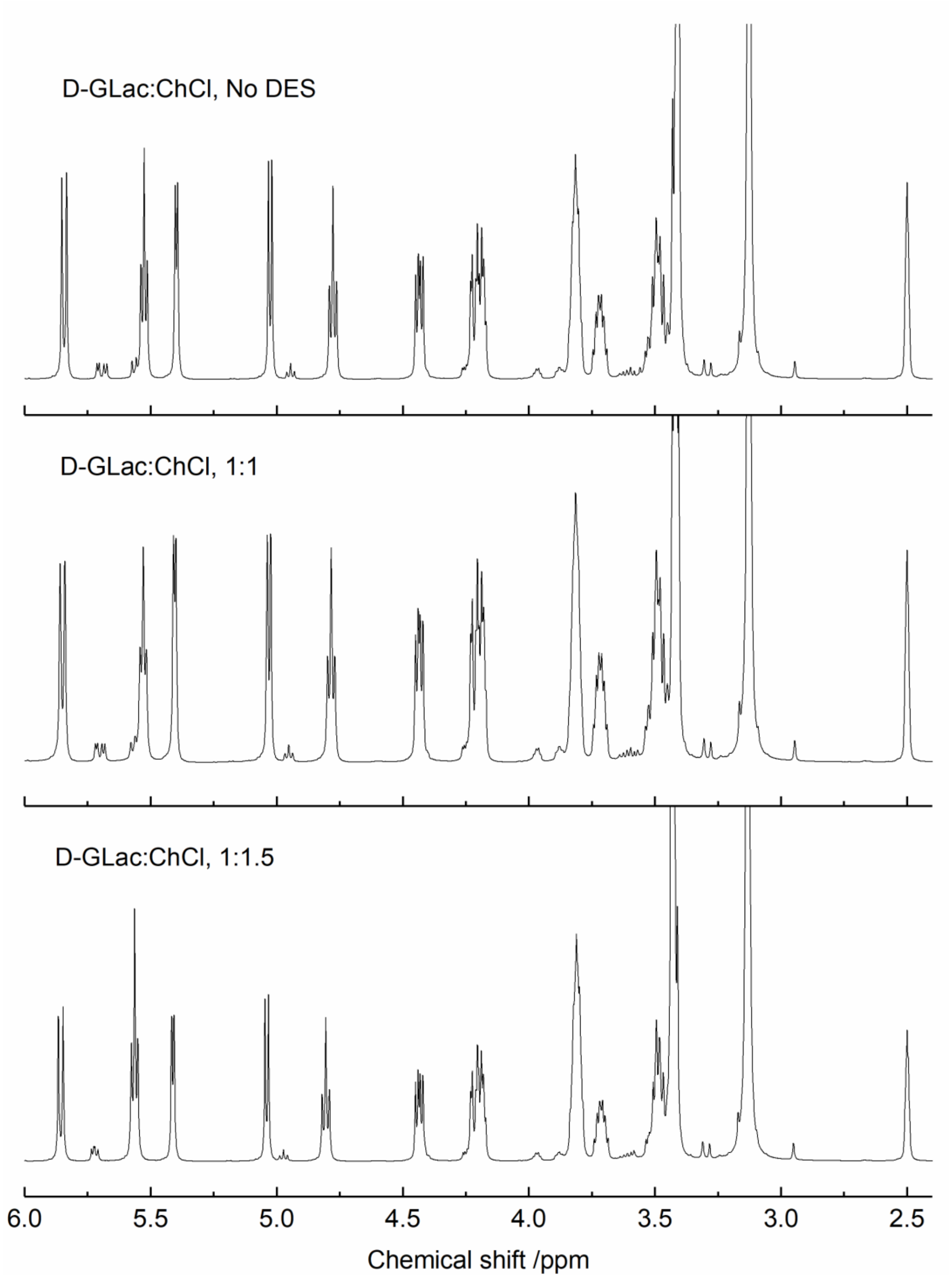

Figure S7. ^{1}H NMR spectra for a mixed solution of D-GLac and ChCl and diluted D-GLac-containing DES (all prepared in DMSO-d_6, ca. 50 mg mL^{-1}).

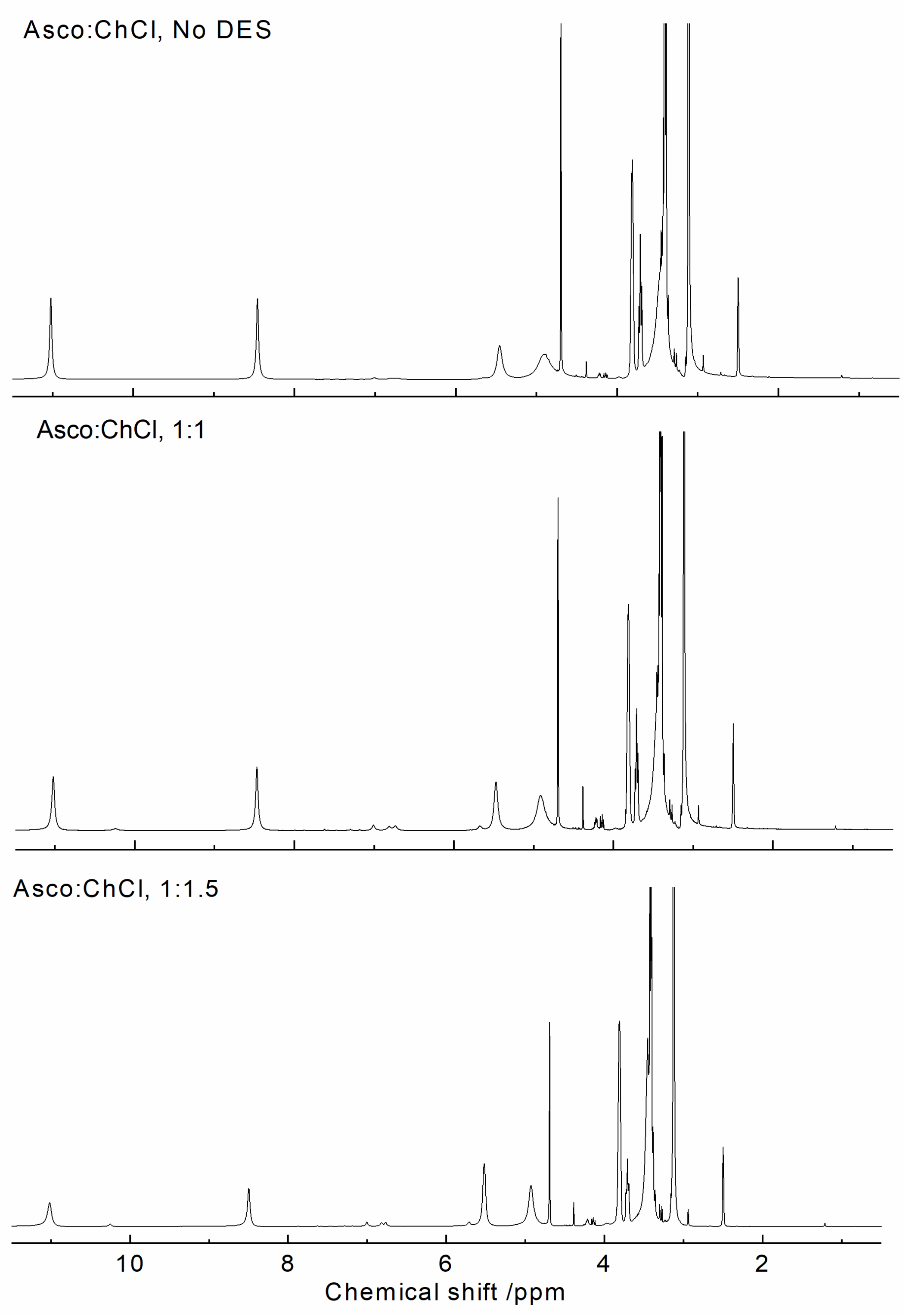

Figure S8. ^{1}H NMR spectra for a mixed solution of L-ascorbic acid and ChCl and diluted L-ascorbic acid-containing DES (all prepared in DMSO-d_6, *ca*. 50 mg mL^{-1}).

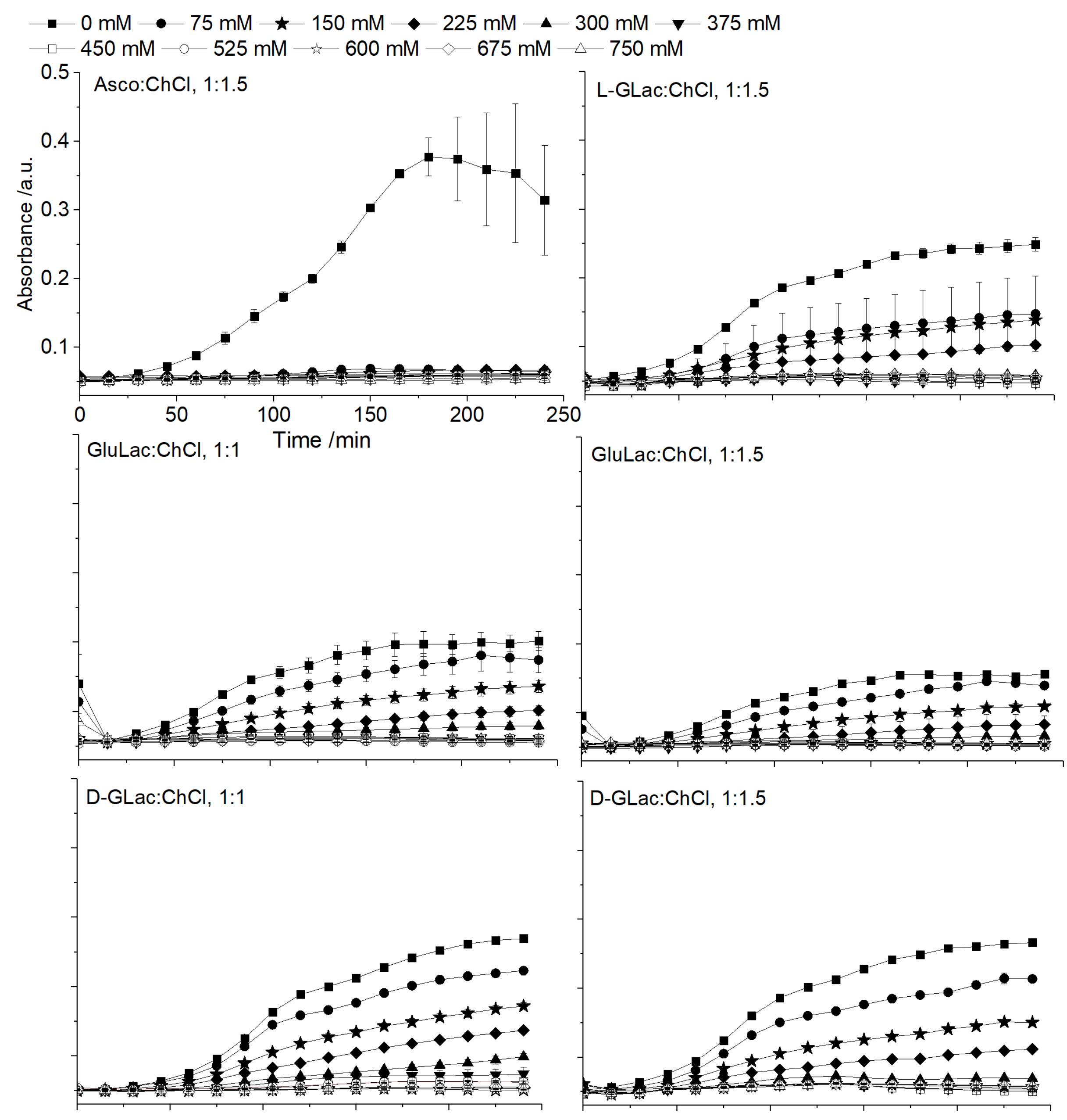

Figure S9. Evolution of optical density of LB growth media containing Asco or lactone-based DES at 0 - 750 mM.

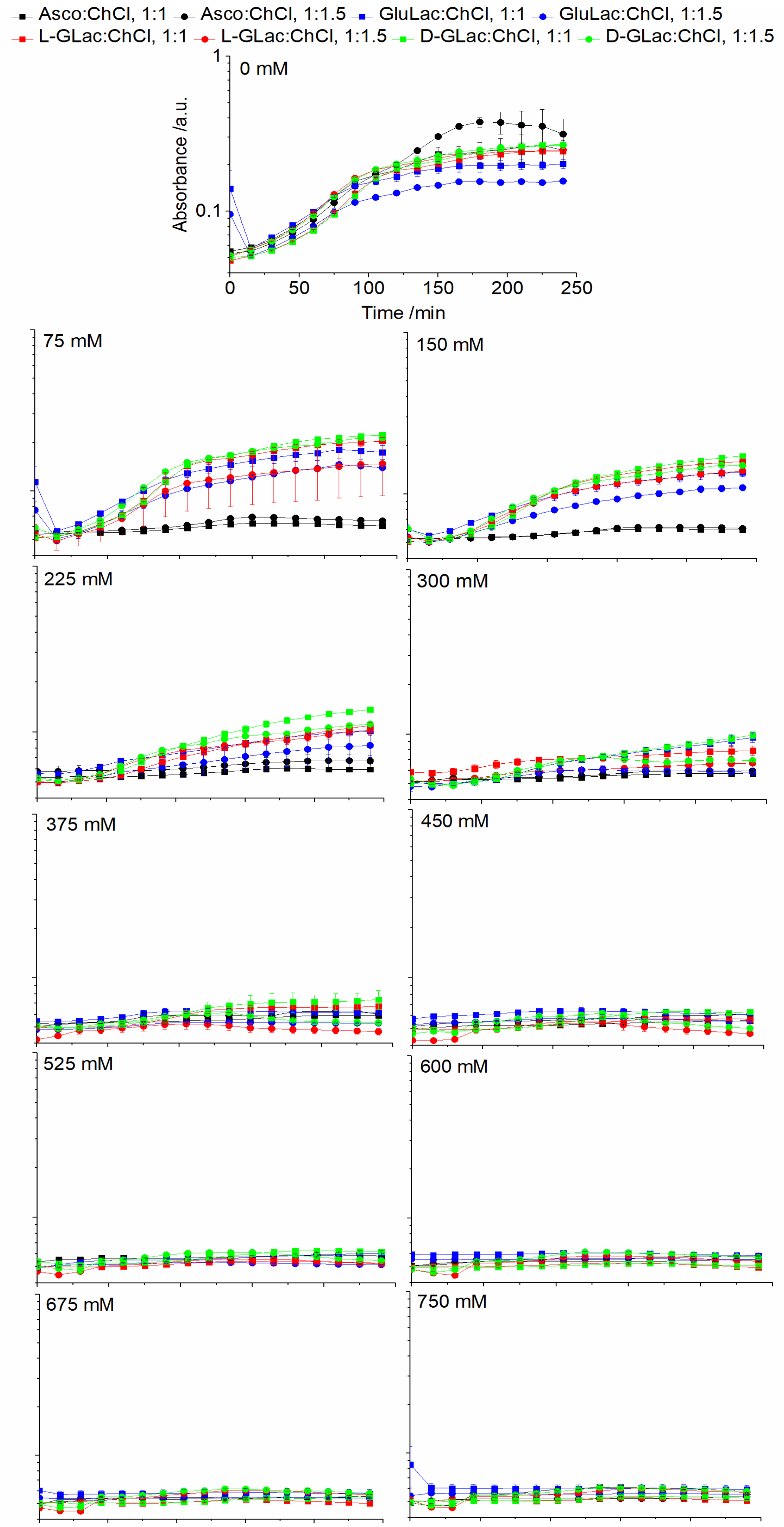

Figure S10. Comparison of optical densities for LB growth media at fixed concentrations of Asco and lactone-based DES.

Note that in some cases there were no data points collected at the lowest shear rates as a reading could not be obtained. This is considered to be an artefact of the experiments given that this was not always repeatable.

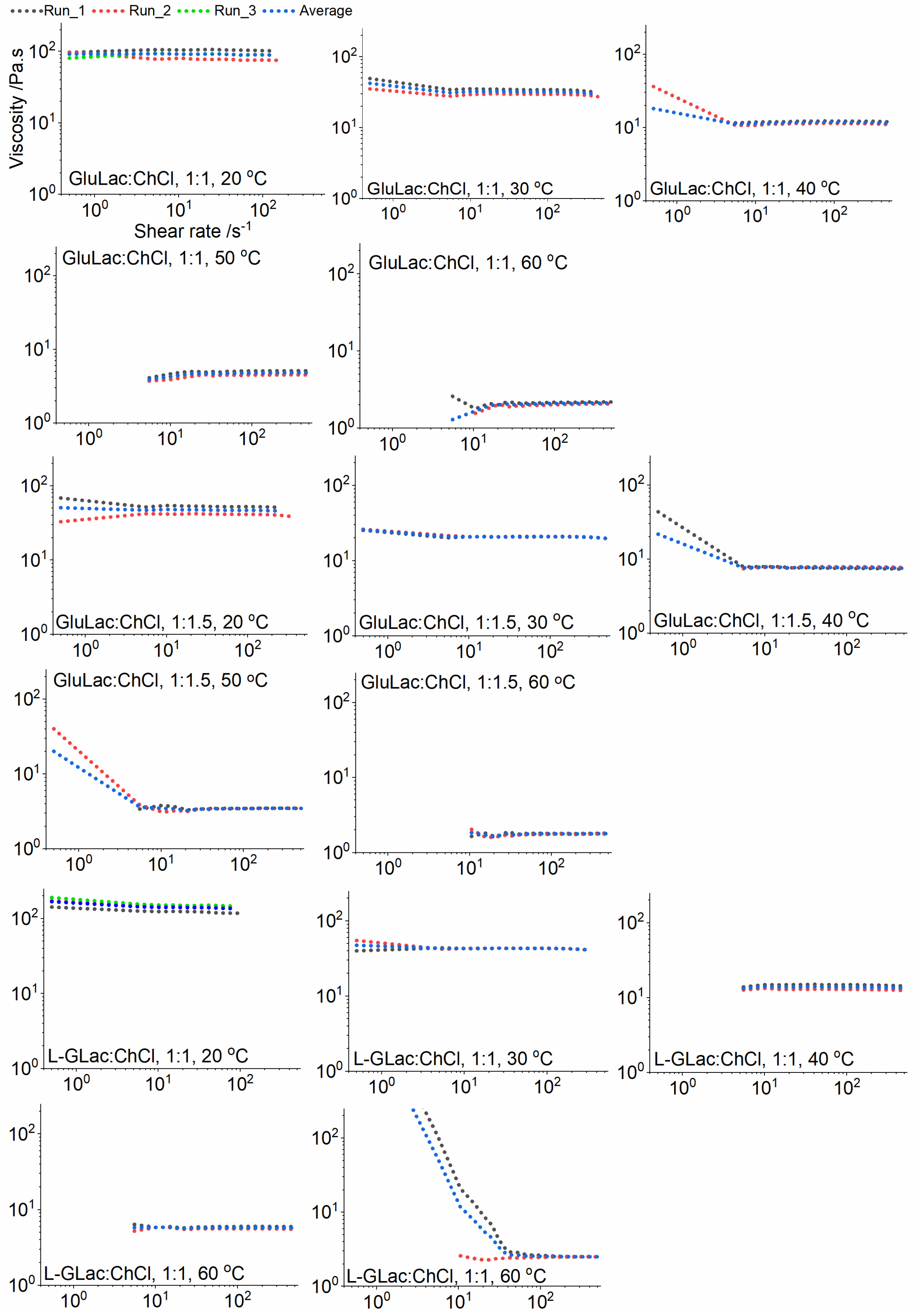

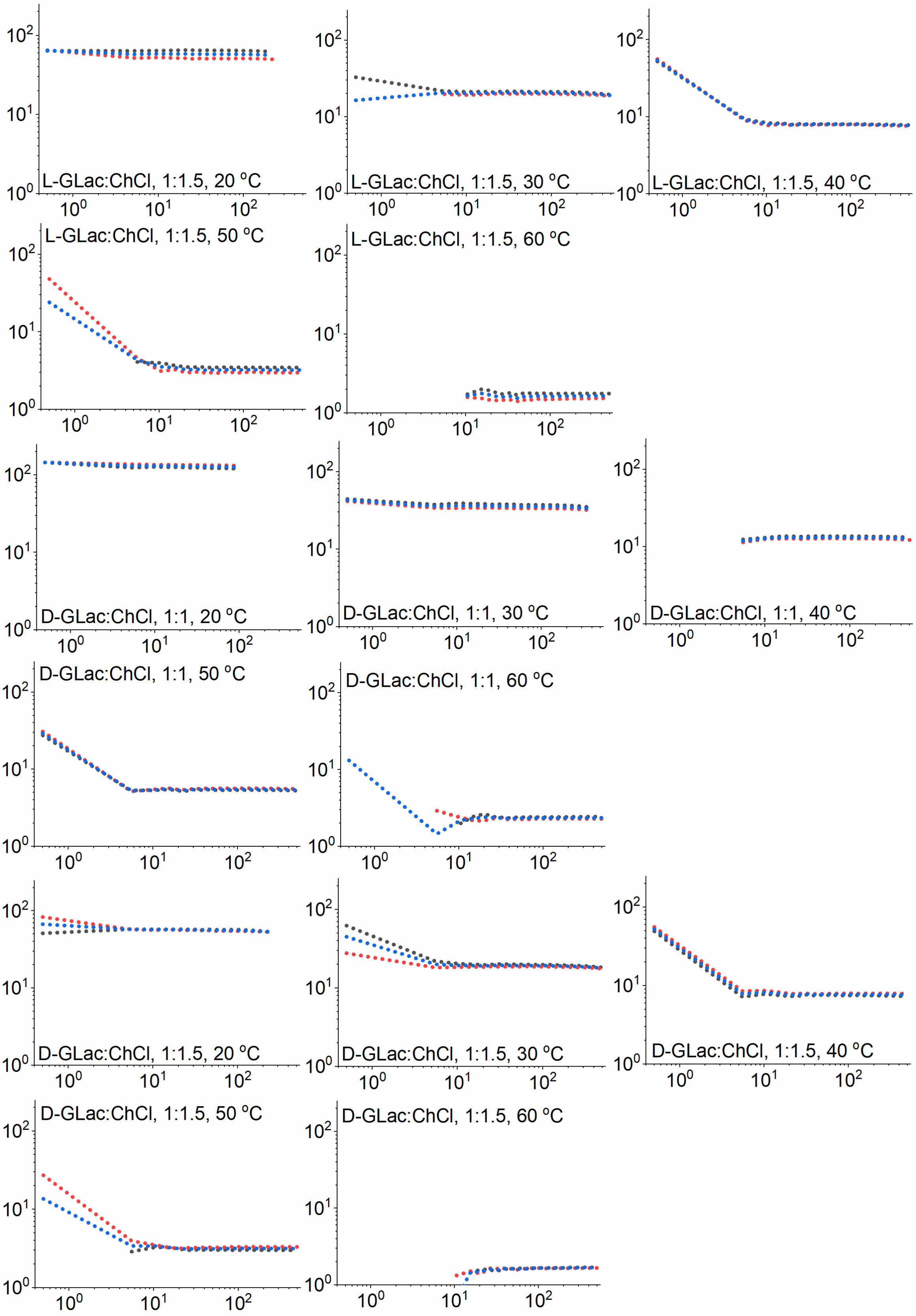
L-GLac:ChCl, 1:1.5, 20 °C
L-GLac:ChCl, 1:1.5, 30 °C
L-GLac:ChCl, 1:1.5, 40 °C
L-GLac:ChCl, 1:1.5, 50 °C
L-GLac:ChCl, 1:1.5, 60 °C
D-GLac:ChCl, 1:1, 20 °C
D-GLac:ChCl, 1:1, 30 °C
D-GLac:ChCl, 1:1, 40 °C
D-GLac:ChCl, 1:1, 50 °C
D-GLac:ChCl, 1:1, 60 °C
D-GLac:ChCl, 1:1.5, 20 °C
D-GLac:ChCl, 1:1.5, 30 °C
D-GLac:ChCl, 1:1.5, 40 °C
D-GLac:ChCl, 1:1.5, 50 °C
D-GLac:ChCl, 1:1.5, 60 °C
10^0
10^1
10^2

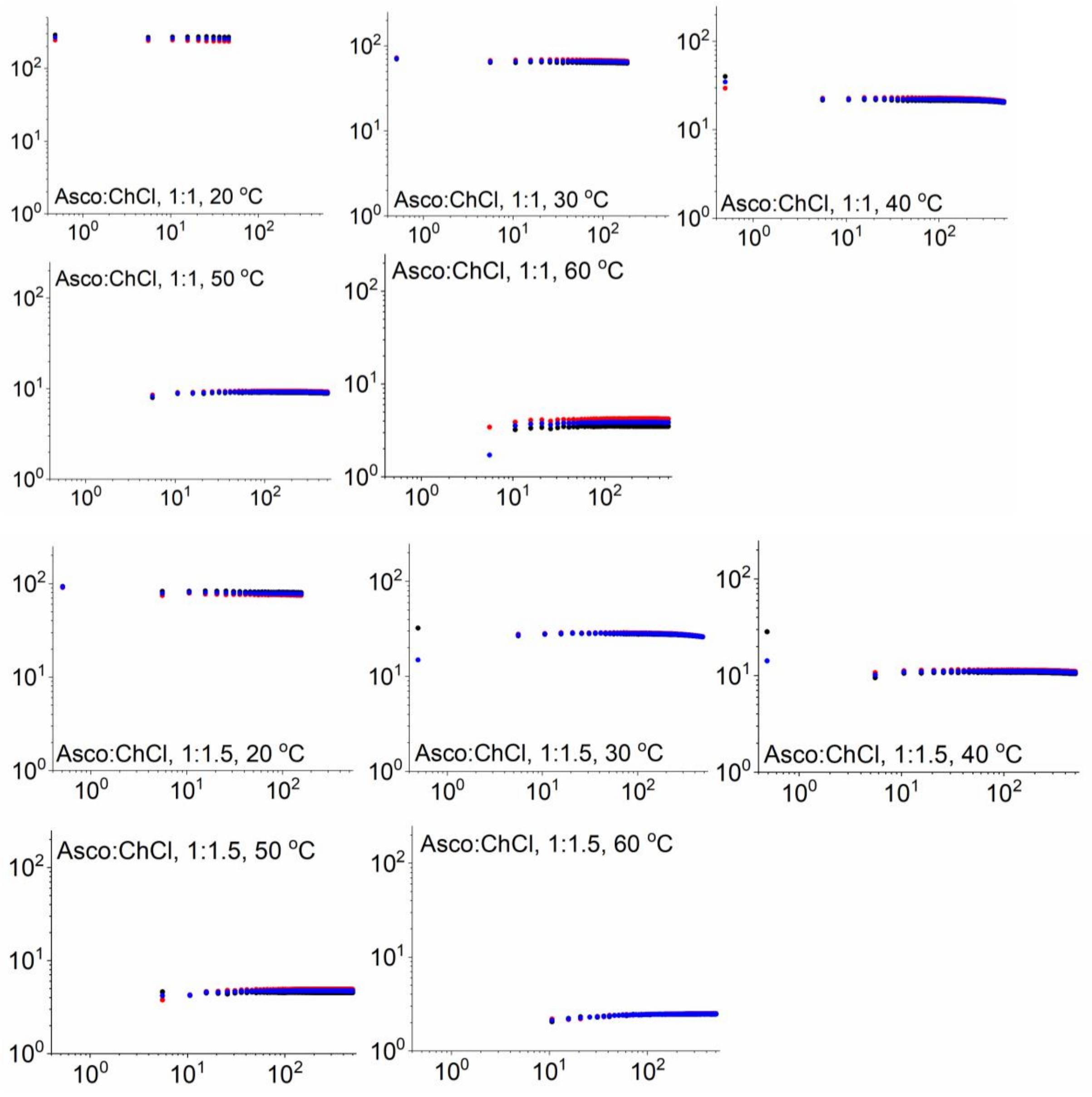

Figure S11. Viscosity of lactone and ascorbic acid-based DES (Pa.s) as a function of shear rate (s^{-1}) from 20 to 60 ^{o}C.

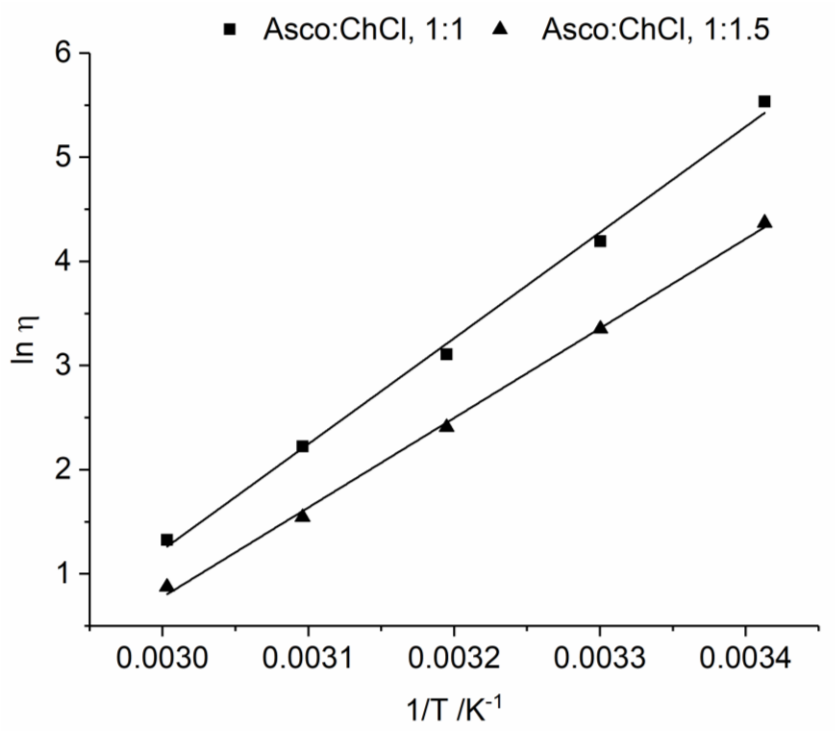

Figure S12. Arrhenius plot for L-ascorbic acid:ChCl DES at 20 to 60 ºC (determined using the viscosity measured at a shear rate of 45 s^{-1} (20 ºC, Asco:ChCl, 1:1) or 50 s^{-1} (30 - 60 ºC, Asco:ChCl, 1:1 and 20 to 60 ºC Asco:ChCl 1:1.5).

Table S2. Parameters for the Arrhenius equation ($\eta 0$, Ea and R^2) of Asco:ChCl DES.

**System, molar ratio**	**η_0 (Pa.s)**	**E_a (kJmol^{-1})**	**R^2**
Asco:ChCl, 1:1	2.012 E-13	84.436	0.996
Asco:ChCl, 1:1.5	1.370 E-11	71.481	0.998
